# Publisher Correction: Two-dimensional Kβ-Kα fluorescence spectrum by nonlinear resonant inelastic X-ray scattering

**DOI:** 10.1038/s41467-023-40664-5

**Published:** 2023-08-10

**Authors:** Kenji Tamasaku, Munetaka Taguchi, Ichiro Inoue, Taito Osaka, Yuichi Inubushi, Makina Yabashi, Tetsuya Ishikawa

**Affiliations:** 1 RIKEN SPring-8 Center, 1-1-1 Kouto, Sayo-cho, Sayo-gun, Hyogo, 679-5148 Japan; 2 Japan Synchrotron Radiation Research Institute, 1-1-1 Kouto, Sayo-cho, Sayo-gun, Hyogo, 679-5198 Japan; 3 Toshiba Nanoanalysis Corporation, 8 Shinsugita-cho, Isogo-ku, Yokohama, Kanagawa 235-8522 Japan

**Keywords:** Atomic and molecular interactions with photons, X-rays

Correction to: *Nature Communications* 10.1038/s41467-023-39967-4, published online 17 July 2023

The original version of this Article contained an error in Equation (2) and used incorrect size of the absolute value symbol.

The correct form of Equation (2) is:

$$\frac{{d}^{2}{\sigma }_{{{{{{\rm{RIXS}}}}}}}({\omega }_{1},{\omega }_{2})} {d\hslash {\omega }_{2}d{\Omega }_{2}}={m}^{2}{r}_{0}^{2}\left(\frac{{\omega }_{2}}{{\omega }_{1}}\right)\mathop{\sum}\limits_{f}{\left|\mathop{\sum}\limits_{n}\frac{{\omega }_{fn}{\omega }_{ni} \left \langle f \left |\hat{r} \right |n \right\rangle \left \langle n \left | \hat{r} \right | i \right \rangle }{\hslash {\omega }_{1}-\hslash {\omega }_{ni} - i {\Gamma }_{n}/2}\right|}^{2} \times \delta \left(\hslash {\omega }_{1} - \hslash {\omega }_{2}-\hslash {\omega }_{fi}\right),$$

This has been corrected in the PDF and HTML versions of the Article.

