# Supplementary material for: Two-dimensional Kβ-Kα fluorescence spectrum by nonlinear resonant inelastic X-ray scattering
Source: Nat Commun. 2023 Jul 17;14:4262. doi: 10.1038/s41467-023-39967-4 (PMC10352240; doi:10.1038/s41467-023-39967-4)
Supplement: Supplementary file 1 — Supplementary Information [file 41467_2023_39967_MOESM1_ESM.pdf]

Supplementary information: Two-dimensional Kβ-Kα fluorescence spectrum by nonlinear resonant inelastic X-ray scattering

Kenji Tamasaku[1,2], Munetaka Taguchi[3], Ichiro Inoue[1], Taito Osaka[1], Yuichi Inubushi[2,1], Makina Yabashi[1,2], Tetsuya Ishikawa[1]

[1]RIKEN SPring-8 Center, 1-1-1 Kouto, Sayo-cho, Sayo-gun, Hyogo 679-5148, Japan. [2]Japan Synchrotron Radiation Research Institute, 1-1-1 Kouto, Sayo-cho, Sayo-gun, Hyogo 679-5198, Japan. [3]Toshiba Nanoanalysis Corporation, 8 Shinsugita-cho, Isogo-ku, Yokohama, Kanagawa 235-8522, Japan.

Supplementary Note 1. Possible mechanism of suppressing population of multi-hole state

Here, we discuss the reason why $\sigma_{\mathrm{RIXS}}^{(2)}$ retains important feature of the Kβ fluorescence spectrum in spite of the Auger cascade. First, we consider the Auger cascade in an atomic model, though it fails to explain our experimental result. For example, when a 2*s* core hole is created (Supplementary Fig. 1), it may decay mainly by an $L_1 \rightarrow L_{2,3}M_{4,5}$ Coster-Kronig transition[1], yielding a $2p^5 3d^9$ state with one 3*d* hole. Then, the 2*p* hole may decay mainly by an $L_{2,3} \rightarrow M_{2,3}M_{4,5}$ Auger transition[2]. These successive transitions, i.e., the Auger cascade chain, creates a $3p^5 3d^8$ state, which can be the initial state of the RIXS part. Some states in the $3p^5 3d^8$ configuration have longer lifetime due to lack of efficient decay process under an isolated condition. When we take into account all possible Auger cascade chains invoked by 2*s*, 2*p* and 3*s* core-hole creation, $3p^5 3d^l$ configurations, where $l < 9$, can have unignorable population. Such $3p^5 3d^l$ states could have produced corresponding spectral features in $\sigma_{\mathrm{RIXS}}^{(2)}$, which are absent in the Kβ fluorescence spectrum.

In case of copper metal, there are other processes[3], such as, electron-electron scattering, and excitation of plasmon or phonon, which relax 3d holes. In addition, the itinerant 3d hole may quickly leave the absorbing atom. On the other hand, the second Auger decay discussed above has a longer lifetime of several femtoseconds[2]. Thus, the first 3d hole created by $L_1 \rightarrow L_{2,3}M_{4,5}$ may no longer exist on the absorbing atom when a 3p hole is created by the second Auger process. We consider that important multi-hole state in the nonlinear RIXS is a two-hole state with one in the 3p orbital created by a single Auger or Coster-Kronig process, and hence, the $3p^5 3d^9$ state is the dominant multi-hole state for copper metal. We note that $3s^1 3p^5$ and $3p^4$ states can be produced by a single process. The emission photon energies for most of these states lies above the diagram transition[4], and easily distinguishable in the emission spectra. However, we couldn't find these contributions in the 2D fluorescence spectrum (Fig.2 and Supplementary Fig. 4), and consider that the $3s^1 3p^5$ and $3p^4$ states are minor.

In general, the final state of the Kβ emission of a $3d^l$ system includes a $3p^5 3d^{l\text{-}1}$ state due to the shake-off process. The same $3p^5 3d^{l\text{-}1}$ state should appear as a dominant multi-hole state in i of the nonlinear RIXS due to the Auger cascade with the fast 3d-hole decay processes. Because of this correspondence, the 2D fluorescence spectrum, $\sigma^{(2)}_{\mathrm{RIXS}}$, can be compared with the Kβ fluorescence spectrum. However, extra 3d holes may survive in complex molecules, because the decay process for the 3d holes in molecules is limited compared to the solids. This could make the relationship between $\sigma^{(2)}_{\mathrm{RIXS}}$ and the Kβ fluorescence spectrum complicated, though that does not matter if we discuss $\sigma^{(2)}_{\mathrm{RIXS}}$ only, e.g., compare $\sigma^{(2)}_{\mathrm{RIXS}}$ of target sample with reference samples. A positive aspect is that the Auger process should be sensitive to the chemical environment of the absorbing atom, such as, the ligand type, the coordination number, and the symmetry, giving additional sensitivity to the electronic and chemical state to the nonlinear RIXS.

Supplementary Figures

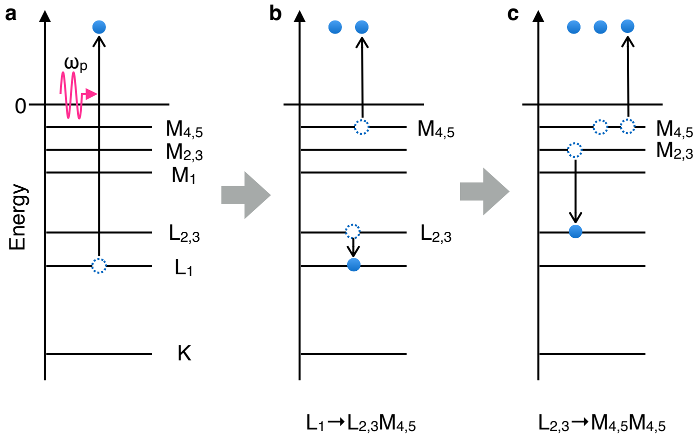

Supplementary Fig. 1: Schematics of main path in Auger cascade initiated by a 2*s* core-hole creation. a. Photoionization of the L_1 subshell. **b.** The $L_1 \rightarrow L_{2,3}M_{4,5}$ Coster-Kronig transition creates a $2p^5 3d^9$ state. **c**. The $L_{2,3} \rightarrow M_{2,3}M_{4,5}$ Auger transition creates a $3p^5 3d^8$ state.

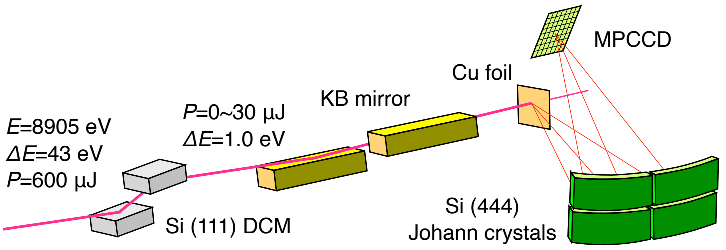

Supplementary Fig. 2: Schematic experimental setup. The beam parameters are indicated as E for the central photon energy, ΔE for the band width and P for the pulse energy. DCM: double-crystal monochromator. KB mirror: Kirkpatrick-Baez mirror. MPCCD: multi-port charge-coupled device.

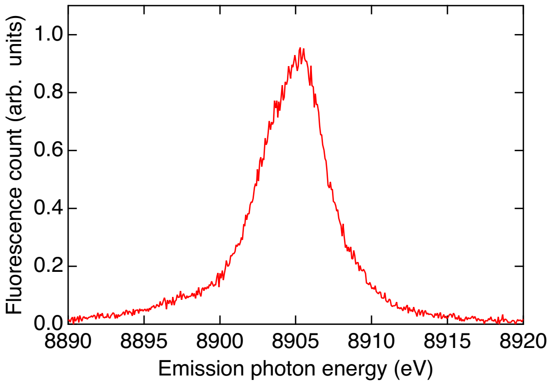

Supplementary Fig. 3: Kβ fluorescence spectrum of copper metal. The fluorescence spectrum was measured by the same single-shot polychromator with three cylindrically bent Si (553) crystals. The dimension of the bent crystals was the same as that described in Methods. The excitation photon energy was 8996 eV just above the absorption edge of copper, and therefore, no satellite peak exists.

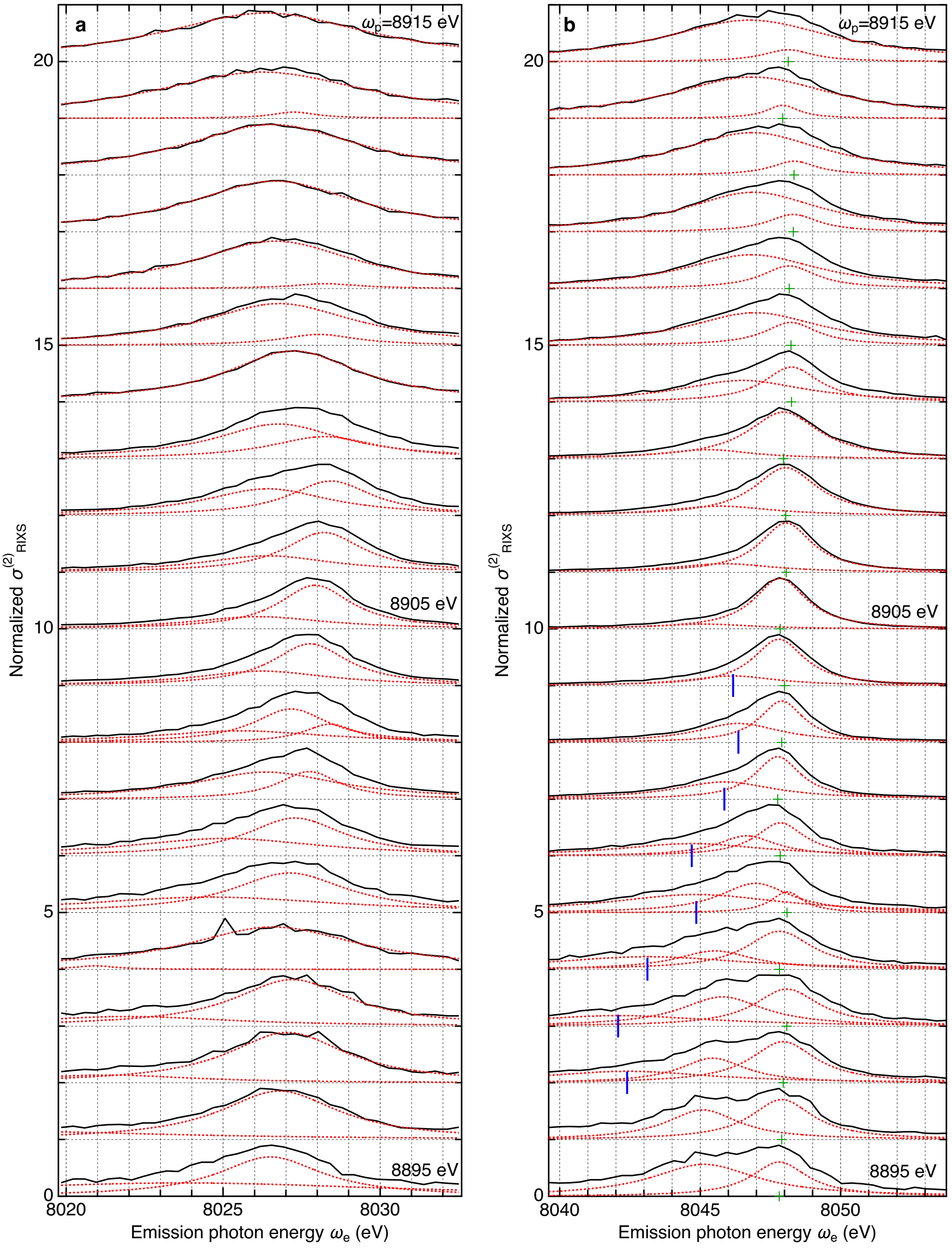

Supplementary Fig. 4: Decomposition of Kα spectrum at constant pump photon energy between 8895 and 8915 eV. a,b, Lorentzian fitting to the emission spectra in the $K\alpha_2$ region (**a**) and $K\alpha_1$ region (**b**). Solid lines represent $\sigma^{(2)}_{\mathrm{RIXS}}$ normalized to its maximum value. Dotted lines are the Lorentzian

components determined by curve fitting. Vertical bars and crosses (**b**) indicate the peak positions for the tail and constant-ω_e components, respectively.

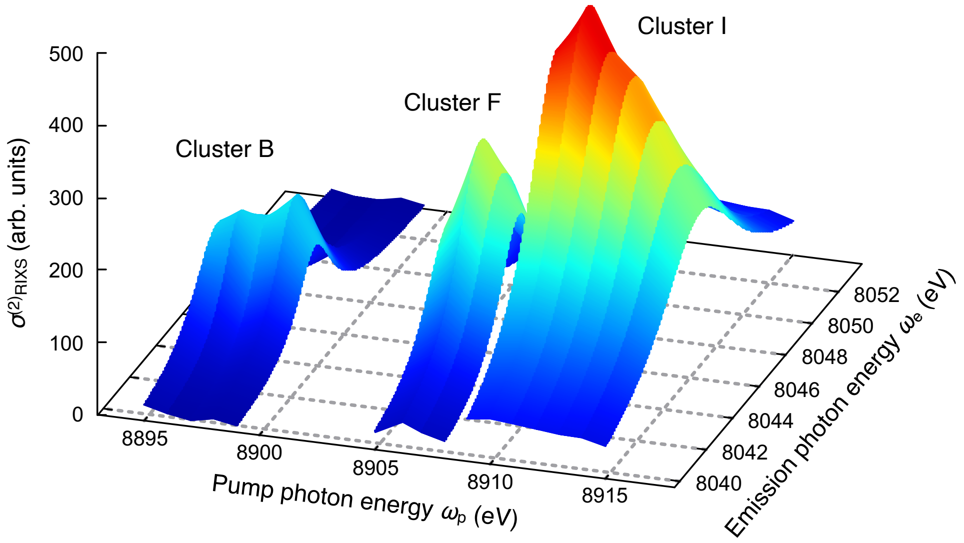

Supplementary Fig. 5: Lorentzian components for $3p^53d^9$ satellite peaks. Lorentzian components in the same cluster are connected. The cluster label is the same as Figs. 2 and 3a.

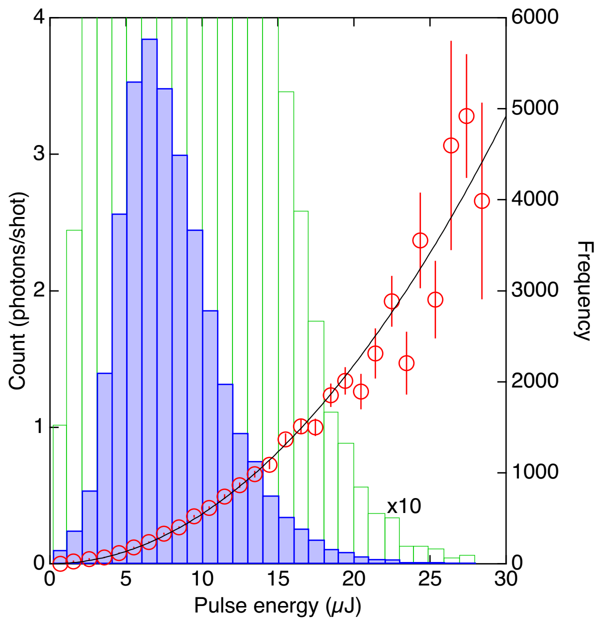

Supplementary Fig. 6: Pulse-energy dependence of 8048-eV emission count rate and histogram of pulse energy at 8905 eV. The emission count rate (open circles with the standard error of the mean) and the fitting curve (solid line) are the same as Fig. 1b except that they are plotted in linear scales. Bars indicate the number of shots for each pulse-energy bin. Open bars are vertically magnified by a factor of ten. Total number of shots was 41609.

Supplementary References

[1] McGuire, E. J. L-Shell Auger and Coster-Kronig Electron Spectra. Phys. Rev. A 3, 1801-1810 (1971).

[2] McGuire, E. J. M-Shell Auger and Coster-Kronig Electron Spectra. Phys. Rev. A 5, 1052-1059 (1972).

[3] Kittel, C. Introduction to Solid State Physics. (John Wiley & Sons, New York, 1986).

[4] Deutsch, M., Hölzer, G., Härtwig, J., Wolf, J., Fritsch, M. & Förster, E. Kα and Kβ x-ray emission spectra of copper. Phys. Rev. A 51, 283-296 (1995).
